# Age-related trajectories of the development of social cognition

**DOI:** 10.3389/fpsyg.2024.1348781

**Published:** 2024-04-22

**Authors:** Zhi-Xiong Yan, Zhe He, Ling-Hui Jiang, Xia Zou

**Affiliations:** ^1^Guangxi Center of Developmental Population Neuroscience, Nanning Normal University, Nanning, China; ^2^Continuing Education School, Guangxi College for Preschool Education, Nanning, China

**Keywords:** age-related trajectories, social cognition, intrinsic functional connectivity, morality, theory of mind, empathy

## Abstract

Age-related trajectories of intrinsic functional connectivity (iFC), which represent the interconnections between discrete regions of the human brain, for processes related to social cognition (SC) provide evidence for social development through neural imaging and can guide clinical interventions when such development is atypical. However, due to the lack of studies investigating brain development over a wide range of ages, the neural mechanisms of SC remain poorly understood, although considerable behavior-related evidence is available. The present study mapped vortex-wise iFC features between SC networks and the entire cerebral cortex by using common functional networks, creating the corresponding age-related trajectories. Three networks [moral cognition, theory of mind (ToM), and empathy] were selected as representative SC networks. The Enhanced Nathan Kline Institute-Rockland Sample (NKI-RS, *N* = 316, ages 8–83 years old) was employed delineate iFC characteristics and construct trajectories. The results showed that the SC networks display unique and overlapping iFC profiles. The iFC of the empathy network, an age-sensitive network, with dorsal attention network was found to exhibit a linear increasing pattern, that of the ventral attention network was observed to exhibit a linear decreasing pattern, and that of the somatomotor and dorsal attention networks was noted to exhibit a quadric-concave iFC pattern. Additionally, a sex-specific effect was observed for the empathy network as it exhibits linear and quadric sex-based differences in iFC with the frontoparietal and vision networks, respectively. The iFC of the ToM network with the ventral attention network exhibits a pronounced quadric-convex (inverted U-shape) trajectory. No linear or quadratic trajectories were noted in the iFC of the moral cognition network. These findings indicate that SC networks exhibit iFC with both low-level (somatomotor, vision) and high-level (attention and control) networks along specific developmental trajectories. The age-related trajectories determined in this study advance our understanding of the neural mechanisms of SC, providing valuable references for identification and intervention in cases of development of atypical SC.

## 1 Introduction

Social cognition (SC) concerns the psychological processes through which individuals interact with others as part of a group (Burnett et al., 2011; Happé et al., 2017). Several structures in the human brain are implicated in SC, such as the medial prefrontal cortex (MPFC), posterior cingulate cortex, temporoparietal junction, and superior temporal sulcus (Adolphs, 2001). These structures are primarily located in the default mode network, a neural network vital to SC (Raichle, 2015). The neural mechanisms of the development of SC in terms of structural developmental trajectories (Mills et al., 2014) have been thoroughly investigated; in addition, the neural mechanisms of development of atypical SC from infancy to adolescence have also been studied (Happé and Frith, 2014). Other studies have focused on the development of SC but have confined to a limited age range or have not employed an SC framework in their analysis (Craik and Bialystok, 2006; Wang, 2012; Yang, 2014; Coupé et al., 2017). Studies of the development of SC over a wide range of ages are thus rare and challenging to conduct, although compelling evidence suggests that SC functions may vary with age (Adolphs, 2001; Frith and Frith, 2008). Thus, a framework of the development of SC may offer a useful method for examining the formation of SC (Zuo et al., 2016).

Large-scale meta-analyses of SC provide evidence of subserved network modules. Bzdok et al. (2012) evaluated 2,607 peak coordinates from 247 experiments and initially identified convergent neural networks related to moral cognition, theory of mind (ToM), and empathy, respectively. Based on 11 social cognitive tasks, Schurz (2021) integrated 188 studies of brain activation and constructed a multilevel model of hierarchical structure. These studies help create the maps of neural networks from which it becomes possible to construct intrinsic functional connectivity (iFC) age-related trajectories of SC networks. As Bzdok's SC maps are beyond specific tasks, the current study applies their maps of SC networks to construct age-related trajectories. The three core SC components illustrated in Bzdok et al. (2012) are introduced below.

Empathy refers to sharing interpersonal experiences and mental states of others (Decety, 2010, 2011). Several studies using behavioral paradigms have explored age-associated empathy over limited age ranges. These studies have shown that infants as young as 14–18 months develop the intention to help others attain goals (Warneken and Tomasello, 2009). This innate empathic response develops into fully fledged empathic ability by adolescence (Fabes et al., 1989; Casey et al., 2005; Paus, 2005). Although scholars generally agree on the mechanisms through which empathy develops in children, the mechanisms underlying its development and display in adults remain controversial. Some studies have suggested that older individuals have a greater capacity for empathy than do younger adults (Happé et al., 1998; Beadle et al., 2015), whereas others have reported that empathy weakens as individuals age (Charlton et al., 2009). An age-related decline was reported in a study using functional magnetic resonance imaging (fMRI) in which participants perceived that an individual was experiencing pain (Chen et al., 2014). Thus, although it is agreed that empathy develops throughout the life span, the exact trajectory of this development in adulthood remains controversial (Duval et al., 2011).

Theory of mind (ToM), defined as the inference of others' thoughts, intentions, or behaviors, is pivotal to the development of SC (Premack and Woodruff, 1978; Flavell, 1999). Several studies have suggested that ToM is developed in infancy and matures in adulthood. Specifically, 1-year-old children can share attention (Leung and Rheingold, 1981), 2-year-old children can distinguish pretenses or desires (Wellman and Woolley, 1990), and children aged approximately 5 years old can distinguish true from false beliefs (Perner and Wimmer, 1985; Zaitchik, 1990). ToM is a set of habits and abilities that develop throughout adolescence and early adulthood (Rieffe et al., 2005; Williams et al., 2009; Rosenberg-Kima and Sadeh, 2010) as cognitive abilities mature (Brunet et al., 2000; Maylor et al., 2002). Older adults may rely on functional interconnectivity to compensate for the aging of brain functional networks (Amodio and Frith, 2006; Moran, 2013). In summary, ToM develops from childhood to old age. However, most studies on ToM have been based on observations of behavior rather than an analysis of the underlying neural mechanisms.

Moral cognition, a crucial component of SC, refers to the judging of others' intentions and behaviors. The development of moral cognition has been widely studied. The most influential framework of moral development is Kohlberg's cognitive stage development theory, which posits that moral cognition develops from lower to higher stages aligning with cognitive development (Kohlberg, 1971). Several developmental studies have suggested that moral cognition progresses in stages in tandem with an individual's maturation. Moral cognition begins to develop in infancy; moral-like intentions (e.g., preferentially interacting with prosocial agents) are strongly present in 6-month-old infants (Hamlin et al., 2007). Another study has shown that infants aged between 14 and 18 months old know how to comfort distressed people and exhibit unrewarded helping behavior (Warneken and Tomasello, 2009). Other studies have demonstrated that the distinctions between truth and lies and a shift in moral judgment from a focus on outcome to a focus on intention occur in children aged approximately 8 years old (Lǎzǎrescu, 2012; Cushman et al., 2013). This shift in moral judgment represents the earliest phase of moral maturity. With experience, early adolescents (aged 11–14 years) learn to separate moral cognition from behavior (i.e., they can consider the morality of actions even in cases where they cannot see or know the outcomes); this development represents the attainment of nearly full moral maturity (Caravita et al., 2014). However, one study reported that activity in the moral-related structures changes with advancing age; a decreased activity in the amygdala and an increased activity in the ventromedial prefrontal cortex were observed in fMRI measurements of individuals aged 4–37 years as they viewed scenarios involving accidental or intentional harm to people and objects (Decety et al., 2012). By contrast, a study on moral development indicated that it is relatively stable from preschool to early adulthood (Lapsley and Carlo, 2014). Moral development is thus a complex and poorly understood process requiring investigation by novel methods to determine its specific chronological phases.

The aforementioned studies have uncovered substantial (albeit inconsistent) behavioral evidence on the development of SC but have not focused on the specific neural processes involved. Moreover, neuroimaging approaches (such as fMRI) have rarely been used to investigate development of SC across a wide age range. Hence, this study aims to map the iFC patterns of three core SC functions (empathy, ToM, and moral cognition) in common cortical networks (Yeo et al., 2011) and construct age-related developmental trajectories by using fMRI datasets (Nooner, 2012).

## 2 Methods

### 2.1 Participants

The resting-state fMRI age-related data used in this study were obtained from the Enhanced Nathan Kline Institute—Rockland Sample (NKI-RS, http://fcon_1000.projects.nitrc.org/indi/enhanced). We collected data from 316 healthy participants (average age 44.38 years; 112 men) aged between 8 and 83 years (Table 1). Participants with psychiatric conditions—schizophrenia, posttraumatic stress disorder, attention-deficit hyperactivity disorder, panic disorder, autism, or other mental health problems—were excluded. This dataset was obtained with the approval of the Nathan Kline Institute (Nooner, 2012).

**Table 1:** Sample characteristics and MRI acquisition protocol in the Nathan Kline Institute—Rockland Sample Enhanced.

**Demography**		
*N*	316	112 Males
Age (years)	44.38 ± 19.72	9–83
	**Anatomical (MPRAGE)**	**Resting functional (Multiband)**
TR	1,900 ms	645 ms
TE	2.52 ms	30 ms
Inversion time	900 ms	
Flip angle	9°	60°
Field of view	250 mm	222 mm
Slices	176	40
Voxel size	1.0 × 1.0 × 1.0 mm	3.0 × 3.0 × 3.0 mm

### 2.2 Image preprocessing

The Connectome Computation System (CCS: https://github.com/zuoxinian/CCS; Xu et al., 2015) was used for image preprocessing, mapping of individual-level and group-level connectomics, trajectory construction, and visualization due to its excellent performance in mapping the cortical mantle.

Structural MRI data preprocessing comprised (1) denoising, skull stripping, and structural segmentation using Volbrain, an automated online brain volumetry system (Manjon and Coupe, 2016); (2) cortical surface reconstruction by using FreeSurfer (http://www.freesurfer.net); and (3) boundary-based registration (BBR) for alignment (Greve and Fischl, 2009). The rs-fMRI image preprocessing mainly comprised (1) denoising and the removal of motion artifacts by using an independent component analysis(ICA)-based strategy for Automatic Removal of Motion Artifacts (ICA-AROMA) (Pruim et al., 2015); (2) removal of the first eight volumes to reach stable scanning; (3) masking using the functional brain mask generated on the basis of both three-dimensional structural brain and four-dimensional individual time series; (4) normalization to achieve a four-dimensional global mean intensity of 10,000 across individuals; (5) regression to remove mean signals from white matter, cerebrospinal fluid, Friston-24 head motion parameters, intracranial volume, and error metrics for BBR; (6) temporal smoothing involving band-pass filtering; (7) detrending by removing both linear and quadratic trends in the time domain; and (8) projection onto a fsaverage surface grid and downsampling onto a fsaverage5 surface grid.

### 2.3 Functional connectivity and trajectory construction

**Figure 1:**
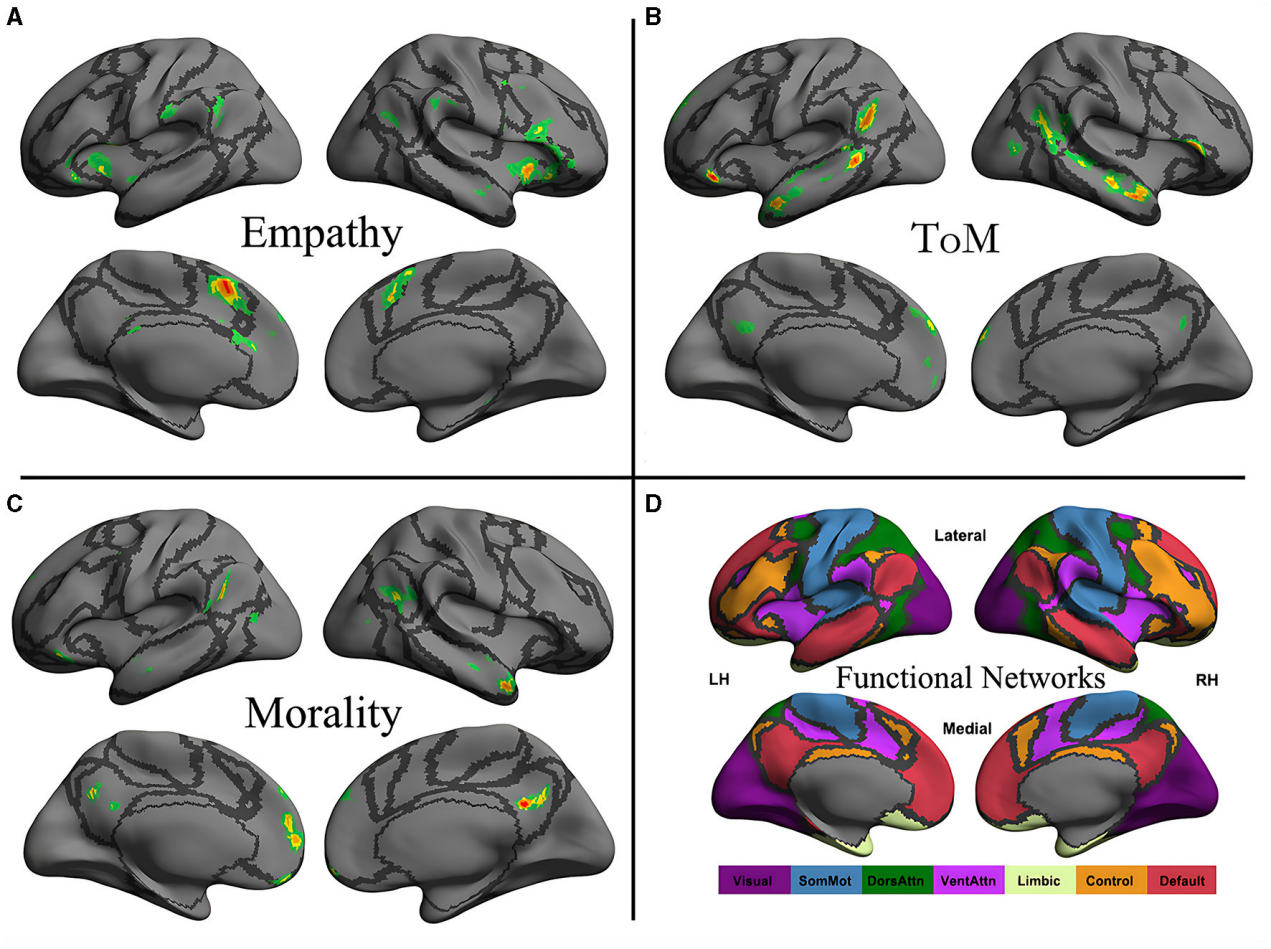
Neural distribution of three social cognitive networks **(A–C)** and seven iFC networks **(D)**. The social cognitive networks are the empathy network **(A)**, the ToM network **(B)**, and the moral cognitive network **(C)**. The seven intrinsic functional networks established by Yeo et al. (2011) using functional parcellation in a sample involving 1,000 healthy participants are the visual (Visual), somatomotor (SomMot), dorsal attention (DorsAttn), ventral attention (VentAttn), limbic (Limbic), frontoparietal control (Control), and default mode (Default) networks. Individual panel plots were constructed for the left and right hemispheres (LH and RH, respectively) and lateral (Lateral) and medial (Medial) views. The cortical grid model “inflated_pre” of the fsaverage function in FreeSurfer was employed to generate the models. Gray curves represent the boundaries of the seven networks.

The original maps for morality, ToM, and empathy networks were obtained from an activation likelihood estimation meta-analysis by Bzdok et al. (2012) and projected to a surface map constructed using the first volume of the images (Figure 1). The boundaries of seven intrinsic functional networks are also presented (Yeo et al., 2011).

The present study used dual regression (DR) to construct individual-level components (Beckmann et al., 2009; Filippini, 2009; Zuo, 2010). This method was based on the following general linear model (GLM) DR equations:


(1) $$\begin{array}{l} X_{ij}^{t}=S^{t}{\left(A_{ij}^{\left(1\right)}\right)}^{t}+{\left(E_{ij}^{\left(1\right)}\right)}^{t},\;X_{ij}=A_{ij}^{\left(1\right)}S_{ij}+E_{ij}^{\left(2\right)},\\ \;1\leq i\leq 3;1\leq j\leq 316 \end{array}$$

In Equation (1), *X*_*ij*_ represents the fMRI data from the *i*-th social network of the *j*-th participant. In the first part of Equation (1), unthresholded group-level components *S* are used as spatial predictors of the individual fMRI volumes and results in the regression matrix $A_{ij}^{\left(1\right)}$ containing the relevant individual regression weights in the time domain (i.e., time series). These time series were then used as temporal predictors for the individual fMRI time series in the second regression equation. The resulting regression matrix *S*_*ij*_ contains regression weights for each component in the spatial domain, where these weights measured network-level functional connectivity (as individual-level DR components). $E_{ij}^{\left(1\right)}$ and $E_{ij}^{\left(2\right)}$ represent the errow terms. These individual-level DR components were subsequently employed to evaluate the agerelated developmental trajectory of group-level components after random field theory (RFT) correction.

To improve the reliability of the trajectories, we applied semiparametric regression models (generalized additive models for location, scale, and shape) to construct trajectories (Rigby et al., 2019) to overcome some of the limitations associated with popular GLMs and generalized additive models.

## 3 Results

**Figure 2:**
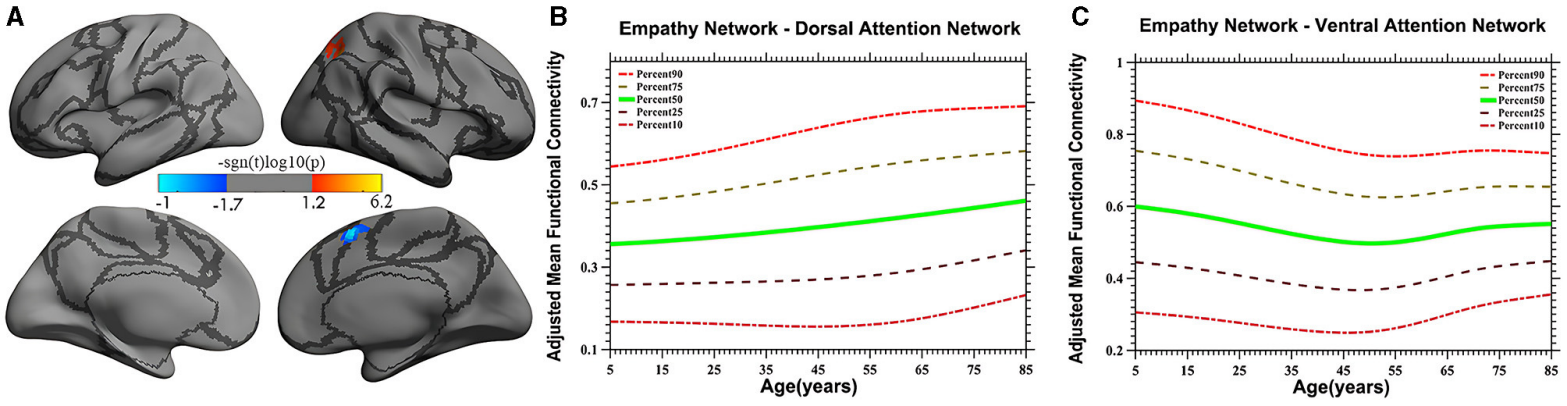
Linear age-related patterns of the iFC of the empathy network in the dorsal attention network [red in **(A)**] and the ventral attention network [blue in **(A)**]. The surface distribution **(A)** was estimated using general linear model (GLM) analysis and random field theory (RFT) correction in FreeSurfer and the Connectome Computation System (CCS). The age-related trajectories were constructed using a semiparametric regression model GAMLSS. The red color bar in panel **(A)** represents a linear increase **(B)**, whereas a linear decrease is shown in blue **(C)**. The five lines in panels **(B, C)** represent the 90th, 75th, 50th, 25th, and 10th percentiles, respectively.

**Figure 3:**
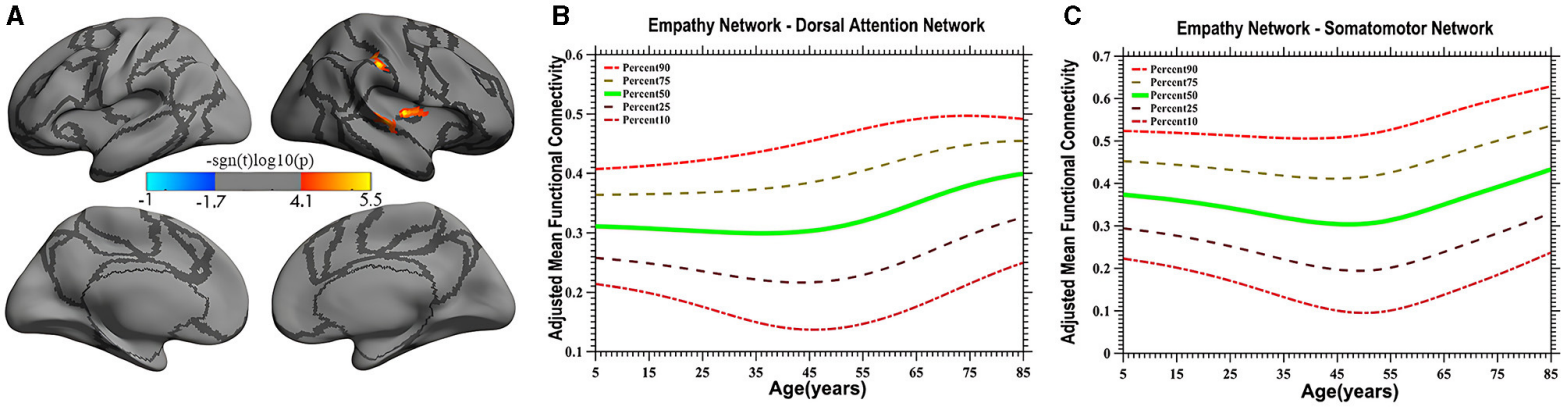
Quadratic pattern of iFC of the empathy network in the dorsal attention and somatomotor networks. The surface distribution **(A)** was estimated using GLM analysis and RFT correction in FreeSurfer and the CCS. The age-related trajectories **(B, C)** were constructed using GAMLSS. Red color **(A)** in the dorsal attention network and somatomotor network indicates an increasing quadratic trend (U-shape), plotted in panels **(B, C)**, respectively. The five lines in panels **(B, C)** represents the 90th, 75th, 50th, 25th, and 10th percentiles, respectively.

**Figure 4:**
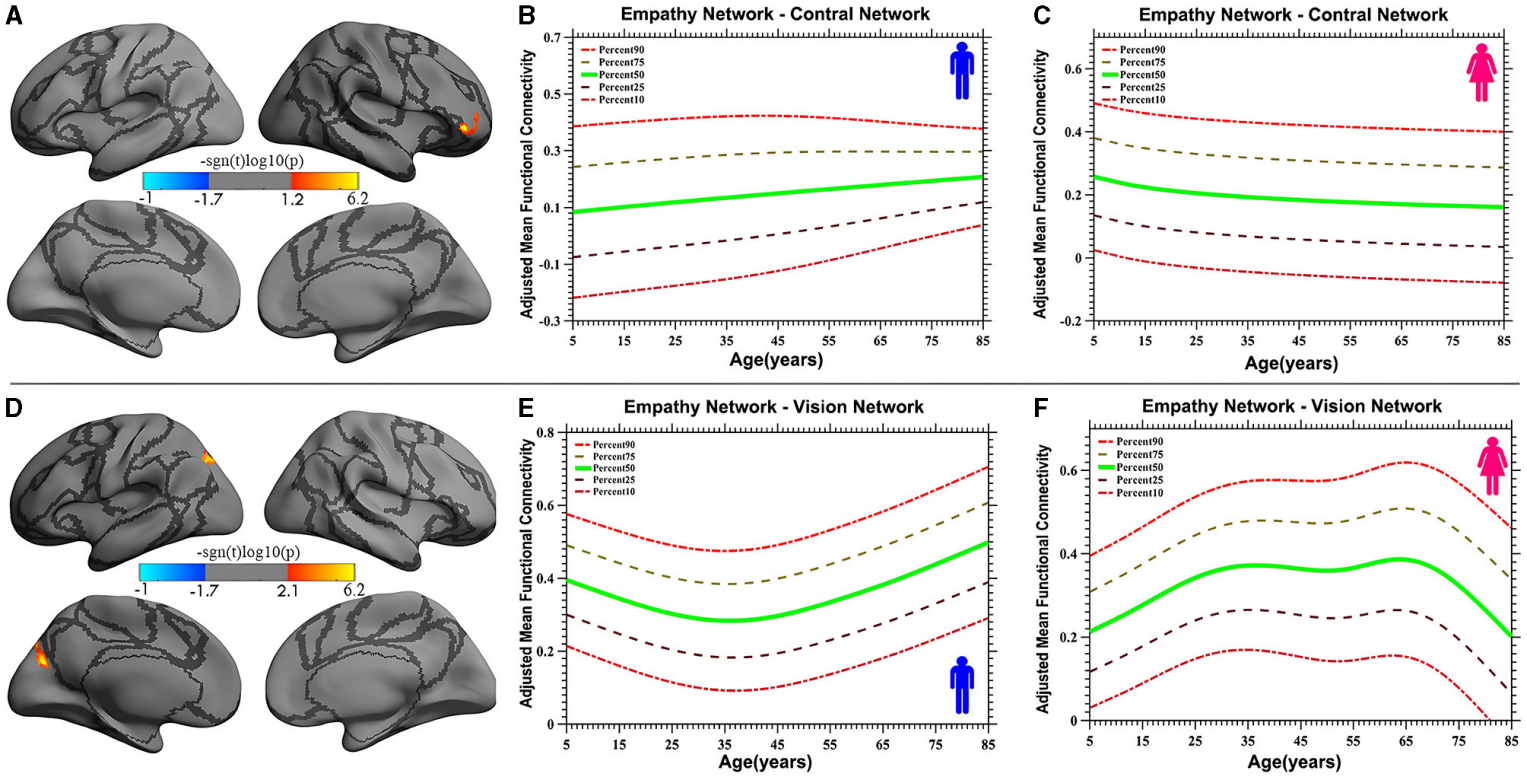
Age-related iFC trajectories of sex-specific effects for the empathy network in the control network [linear, plot **(A)**] and vision network [quadratic, plot **(D)**]. The surface distribution **(A, D)** was estimated using GLM analysis and RFT correction in FreeSurfer and the CCS. The age-related trajectories **(B, C, E, F)** were constructed using GAMLSS. Panels **(B, C, E, F)** indicate the trends of trajectories for men and women, respectively. The five lines in panels **(B, C, E, F)** represent the 90th, 75th, 50th, 25th, and 10th percentiles, respectively.

**Figure 5:**
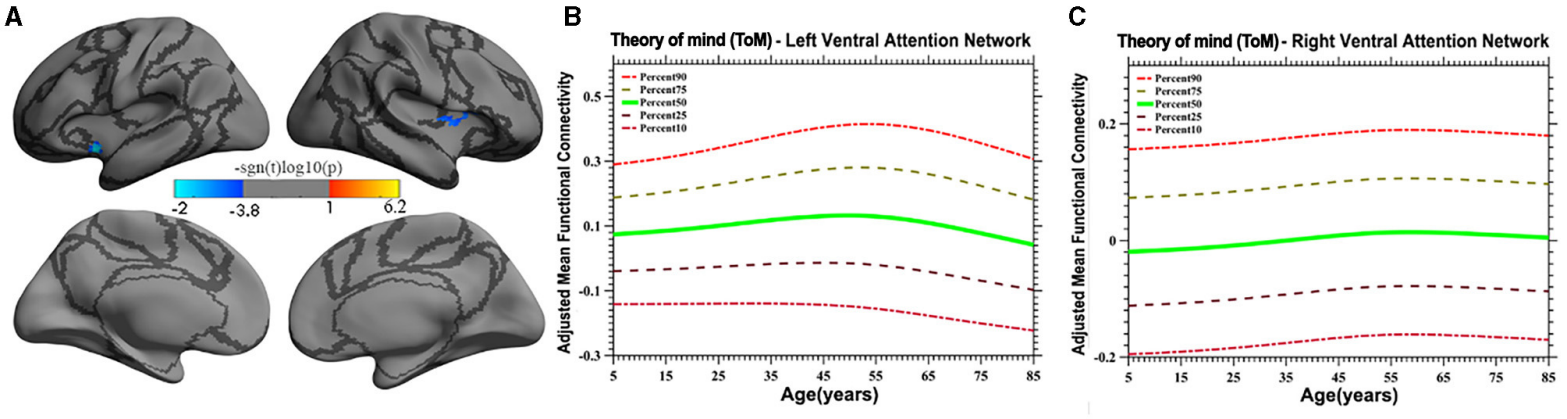
Age-related quadratic iFC trajectories of the ToM network in the left and right ventral attention network. The surface distribution **(A)** was estimated using GLM and RFT correction in FreeSurfer and the CCS. The age-related trajectories **(B, C)** were constructed using GAMLSS. Panels **(B, C)** plot the trajectory of the iFC over the left and right ventral attention network, respectively. The five lines in panels B and C represent the 90th, 75th, 50th, 25th, and 10th percentiles, respectively.

Vertex-based iFC and age-related changes in SC networks and common functional networks were analyzed. All the SC networks except the moral cognitive network exhibited significant age-related variance. The empathy network was the most complex of the SC networks, exhibiting both linear and inverted U-shaped age-related trajectories (Figures 2, 3). Specifically, a linearly increasing iFC was found between the empathy and dorsal attention networks, whereas a linearly decreasing iFC was observed between the empathy and ventral attention networks (Figure 2). Moreover, a quadratic-convex (inverted U-shaped) iFC pattern was noted in the somatomotor and dorsal attention networks (Figure 3). Sex-based effects were determined in the iFC over the empathy and frontoparietal control networks, with men exhibiting greater iFC than women (Figure 4). A further interaction between sex-related and age-related quadratic trends were found over the empathy network and visions network (Figure 4), in which the interaction for men exhibited a quadratic-concave (U-shape) trend, whereas the interaction for women exhibited a quadric-convex (inverted U-shape) trend. Additionally, age-related changes in the iFC of the ToM network took the form of an inverted U-shaped trajectory over the left and right ventral attention networks only (Figure 5).

## 4 Discussion

The results of the present study reveal that the three SC networks have extensive iFC over the cerebral cortex networks (the vision, somatomotor, ventral attention, dorsal attention, and control networks) and exhibit linear and quadratic iFC trends over a wide range of ages. The iFC of the empathy network with the dorsal attention and somatomotor networks exhibits greater age-related variation (linear increases and decreases with an inverted U-shape) than the iFC of the ToM and moral networks. The iFC of the ToM network with a ventral attention network exhibits a quadratic-convex trajectory. Moreover, the empathy network exhibits a sex-dependent effect. On the basis of these findings, we suggest that SC has distinct developmental profiles and functional connectivity properties at various stages of the human life span.

Large-scale functional networks are valuable tools for exploring neuronal mechanisms, and several rs-fMRI studies have demonstrated that the development of functional networks is relatively stable across the life span, although the microstructure and function of the brain change over time (Fair, 2009; Fransson et al., 2011). This stability suggests that the characteristics of the iFC of SC with other functional networks can be considered to construct developmental trajectories over a wide range of ages although the dataset is in a cross-section design. These findings can be used to monitor development and provide reference for clinical interventions.

### 4.1 iFC trajectories of the empathy network

The iFC of the empathy network exhibits the greatest variety in developmental profiles, including both linear and non-linear trajectories. For example, a linearly increasing trend was discovered in the dorsal attention network (Figure 2). Studies have demonstrated that the dorsal attention network involves top-down regulation (Fox, 2006; Koziol et al., 2014). The increasing iFC with the dorsal attention network may indicate that human displays of empathy continuously strengthen top-down monitoring as experience is accumulated. These results are inconsistent with those of a study by Greimel et al. (2010), who observed an age-related increase in activity in the fusiform gyrus and inferior frontal gyrus, which are mainly located in the control network. The reason for this discrepancy may be the limited age range (8–27 years) of the participants, which was insufficient to create a developmental profile. Moreover, a study on empathy-related behavior suggested that, compared with a younger group, older adults did not exhibit impaired state-related cognitive empathy but scored higher on subtests of state-affective empathy and exhibited more emotionally empathy behaviors, indicative of stronger top-down processing (Ze et al., 2014). However, this suggestion requires verification in further studies.

In the present study, a linearly decreasing trend was also noted in iFC in the ventral attention network. Studies have shown that the ventral attention network is predominantly implicated in stimulus-driven attentional control field (Vossel et al., 2014) and is vital to bottom-up processing (Etkin et al., 2011). The pronounced linear decrease in the iFC pattern observed in the present study demonstrates that empathy-related bottom-up processing exhibits a downstream profile, suggesting that external stimuli may lose their ability to influence iFC over time. Moreover, a study by Chen et al. (2014) validates the present study's results, revealing an age-related decline in iFC between the anterior insula and the anterior mid-cingulate cortex, areas that partly overlap the areas discovered in the present study. The evidence from the study by Chen et al. (2014) and the present study's age-related trajectories together suggest that increased top-down and decreased bottom-up processing patterns reflect distinct age-related variation in the cognitive strategies used to convey empathy.

In the present study, a quadratic-concave (U-shape) age-related change in the empathy network's iFC with the somatomotor network and the inferior portion of the dorsal attention network was noted (Figure 3A), indicating that empathy is predominantly associated with sensory-motor function and top-down processing. Several studies have implicated somatosensory regions (e.g., the postcentral cortex) as likely candidates for encoding social perception (Meyer et al., 2011; Morrison, 2013). These results suggest that the brain's somatosensory regions encode socially relevant stimuli, consistent with the hypothesis that socially derived information is acquired by mentally mirroring the behavior of others (Keysers and Gazzola, 2010). The present study extends the results of the literature by revealing distinct developmental characteristics reflective of efficient iFC with the somatosensory and ventral portions of the dorsal attention networks in adults (individuals 40–50 years old) compared with children and older individuals, although exactly what constitutes iFC remains controversial (Power et al., 2010).

The study also discovered a sex-specific difference in iFC between the empathy network and functional networks. Specifically, a trend of linear development was detected between the empathy and control networks, in which the trend for men was a linear increase whereas that for women was a linear decrease. Quadratic trajectories were also found in the vision network, with men having a U-shaped trajectory and women having an inverted U-shaped trajectory. Numerous studies have investigated sex-based differences in empathy development, with most indicating that women are more empathic than men (Christov-Moore et al., 2014). These studies have focused on behavioral and self-reported differences. However, one neuroimaging study revealed no sex-related differences in hemodynamic responses (Michalska et al., 2013). The limited number of participants and specific experimental paradigms may have contributed to the lack of observed differences in that study. The present study used the results of a meta-analysis and extended the age range of participants to encompass almost the entire human age range (7–85 years). The linear sex-based difference in the iFC of the empathy network with the control network indicates that men exhibit increasing iFC relative to women, a finding that requires validation with more direct evidence in additional studies. Moreover, an interaction between sex and a quadratic curve trend was found in the iFC of the empathy network with the vision network (the occipital-parietal junction), demonstrating that men display a quadratic-concave iFC pattern and women a quadratic-convex iFC pattern. This result is partly inconsistent with the findings of the study by Tomasi and Volkow (2012), which indicated that women have lower density of functional connectivity in the vision and other sensory networks than do men as they age but that the interaction between age and sex was not significant. One reason for this discrepancy may be the different focus of that study, which examined the default mode network, whereas the present study examined SC networks. This difference also suggests that the SC networks are unusual, with specific sex-based differences in which, compared with children and older individuals, adult women have stronger iFC in the vision network whereas men do not. These sex differences are reflected not only in primary sensory processing networks, such as the vision networks, but also in high-level cognitive processing networks, such as the frontal control networks.

### 4.2 iFC trajectory of the ToM network

The present study discovered that the iFC of the ToM network with the ventral attention network has an inverted U-shaped age-related trajectory. A prior meta-analysis of ToM development suggested a linear increase in the iFC of the ToM for the age group of 3–10 years (Wellman et al., 2001), a finding consistent with the results of the present study for those ages. Other behavioral studies have found similar developmental trends. Children aged 6 or 7 years were able to pass a “belief about belief” test, whereas children aged 9–11 years displayed complex social skills, such as recognizing a social *faux pas* or incorrect behavior. Adolescents more frequently experience self-consciousness, learn new skills, demonstrate flexibility, and use social comparison as a method of self-evaluation than do preadolescents, indicating a growing understanding of the mental state of others (Dumontheil et al., 2010; Vetter et al., 2013). Conversely, aging is associated with impaired social understanding (Moran, 2013). Neuroimaging studies on ToM development are relatively rare. A study on iFC related to the development of the ToM network in children and adolescents revealed that the middle and inferior temporal cortex and anterior cingulate cortex—critical nodes of the ToM network—are linked to the anterior insula of the ventral attention network, which may be responsible for SC (Cauda et al., 2011). As individual age, brain activity in the ToM network (e.g., the MPFC) shifts from the ventral to the dorsal portion of the network, which is associated with maturation of the prefrontal cortex and the development of cognitive functions (Moriguchi, 2007; Sebastian et al., 2012). In summary, the results of the present study extend the literature to cover a wide range of ages and indicated that the trajectory of the iFC of the ToM has an inverted U-shape. However, this conclusion must be treated with due cautious because of the lack of direct evidence.

### 4.3 iFC trajectory of the moral cognition network

Moral cognition is more complex of the three crucial SC components and involves more neutral regions than the ToM or empathy network (Moran, 2013; Greene, 2015). The moral cognitive network is extensively distributed, including in the ventromedial prefrontal cortex/dorsomedial prefrontal cortex, precuneus, temporoparietal junction, posterior cingulate cortex, temporal pole, and middle temporal gyrus (Marazziti, 2013). These structures are predominantly situated at the interface of the ToM and empathy networks, enabling both rational and emotional processing (Bzdok et al., 2012). The results of the present study suggest that these mixed and overlapping neural structures may moderate developmental trends, resulting in less robust age-related iFC changes. These results are supported by a prior longitudinal study showing that regions related to moral cognition (such as the prefrontal, temporal, and parietal cortices) are relatively stable over the life span (Crone and Elzinga, 2015). More sensitive fitting methods and a wider age range of participants are required to verify these findings in future longitudinal studies.

### 4.4 Applications of age-related developmental trajectories of social cognition

Age-related trajectories of development of SC elucidate the process of normal brain growth and aid in the construction of normative SC models. The present study offers insights applicable to childhood education and the maintenance of brain function in older adults. Furthermore, the results of the present study may assist researchers in identifying clinical biomarkers of social cognitive disorder, indicating what is typical and atypical to aid in the design of suitable interventions. The trajectories of development of SC also provide detailed information regarding the suitable timing of interventions. For example, adolescence is often viewed as the ideal developmental phase for many interventions because of the vulnerability of adolescents to adverse psychiatric conditions and brain dysfunction (Kadosh et al., 2013; Somerville, 2013). However, the present study demonstrates that SC matures at approximately 35–45 years, well beyond puberty. This later timeframe may represent a stage where interventions that are ineffective in adolescents can be fruitfully employed. By charting the neurodevelopment of SC across a wide range of ages, the present study proposes a holistic view of development of SC to enable early identification of atypical SC growth and treatment for various SC-related pathologies.

### 4.5 Limitations

This study has several limitations. First, SC encompasses many poorly understood but related subcomponent fields (Happé et al., 2017). The present study examined only three core subcomponents to create SC age-related trajectories; other associated elements (e.g., imitation and social exclusion) were beyond its scope. Second, the iFC between the SC networks and common functional networks centered on the cerebral cortex. The role of subcortical structures, such as the amygdala, in the development of SC must be examined in in future studies. Third, age-related social cognitive changes in cross-sectional samples may reflect cohorts effects rather than age effects, which may have adversely affected the calculations of age-related developmental trajectories in the present study. Longitudinal cohort fMRI data is expected to be generated in the future.

## 5 Conclusion

This study provides the first heterogeneous, iFC-based developmental trajectory of the SC networks at the vertex level. Specific age-related changes in profiles during the development of SC over a wide age range were observed. The empathy network was found to have an especially complex profile. Linearly decreasing iFC and linearly increasing iFC with the dorsal attention network and ventral attention network, respectively, were found, whereas the trajectory was U-shaped for iFC with somatomotor and dorsal attention networks. Moreover, increasingly linear and U-shaped sex-specific trajectories with the control and vision networks, respectively, were found. The age-related profile of the ToM network exhibits an inverted U-shaped trajectory. These crucial age-related trajectories extend the literature to cover a wide age range and provide a starting point for investigating the extraordinary versatility of the developing social brain, supporting early identification and intervention in cases of atypical development of SC.

## Data availability statement

The original contributions presented in the study are included in the article/supplementary material, further inquiries can be directed to the corresponding author.

## Ethics statement

The studies involving humans were approved by the Ethics Committee of Nanning Normal University. The studies were conducted in accordance with the local legislation and institutional requirements. Written informed consent for participation was not required from the participants or the participants' legal guardians/next of kin in accordance with the national legislation and institutional requirements.

## Author contributions

Z-XY: Writing – original draft, Visualization, Supervision, Formal analysis. ZH: Writing – original draft, Investigation, Formal analysis. L-HJ: Writing – original draft, Visualization, Data curation. XZ: Writing – review & editing, Supervision.
